# Experiences of Fixed Oral Rehabilitation in Older Persons — Motivation, Procedural Strains and Rewarding Improvements

**DOI:** 10.1111/joor.70283

**Published:** 2026-09-07

**Authors:** L. Hedberg, C. Elleby, M. Trulsson, A. Kumar, U. Ekman, B. Cronfalk Seiger, P. Skott

**Affiliations:** ^1^ Public Dental Services Stockholm Sweden; ^2^ Academic Center for Geriatric Dentistry Stockholm Sweden; ^3^ Department of Dental Medicine Karolinska Institutet Huddinge Sweden; ^4^ Department of Conservative Dentistry and Endodontics, Dr. D. Y. Patil Dental College & Hospital Dr. D. Y. Patil Vidyapeeth (Deemed To Be University) Pune India; ^5^ Women's Health and Allied Health Professionals Theme, Medical Unit Allied Health Professionals Karolinska University Hospital Stockholm Sweden; ^6^ Department of Neurobiology, Care Sciences and Society, Division of Clinical Geriatrics Karolinska Institutet Stockholm Sweden

**Keywords:** aged, mastication, oral rehabilitation, person‐centred care, qualitative research, quality of life, tooth loss

## Abstract

**Background:**

Several age‐related changes contribute to deteriorating oral health and risk of tooth loss, with negative effects on oral function and physical and psychological well‐being. To improve these aspects, fixed oral rehabilitation can be offered and has been indicated to also positively impact biopsychosocial parameters.

**Objective:**

To explore how older adults with poor oral function experience their life situation, before, during, and after fixed oral rehabilitation, and their involvement in this therapeutic process.

**Method:**

A qualitative interview study with explorative design included eight older adults from a large intervention study of oral rehabilitation, all ≥ 65 years with an Eichner index <B2. Data were analysed with manifest content analysis.

**Results:**

Our findings suggest that the participants had developed coping strategies regarding eating habits and experienced social detachment and emotional loneliness prior to the rehabilitation. During the oral rehabilitation, the person‐centred approach by the dental team was highly valued, suggesting the need for individualized, tailored support. After completing the oral rehabilitation, a sense of relief, self‐worth, satisfaction, and self‐mastery were emphasised by the participants in addition to the obvious improvement in chewing ability. An unexpected finding following the oral rehabilitation was the participants' lack of post‐care initiatives.

**Conclusions:**

This qualitative study emphasises the impact of poor oral health in older adults, also concerning their emotional well‐being, before oral rehabilitation. To improve older persons' oral health and function late in life, we suggest a tailored person‐centred approach during their oral rehabilitation, but also structured referral and dental care strategies post oral rehabilitation.

**Trail Registration:**

ClinicalTrials.gov identifier: NCT06783426.

## Graphical abstract

In this study, eight older adults with poor oral function were interviewed to explore their experience of life before, during, and after fixed oral rehabilitation. A descriptive qualitative content analysis method was employed. Three key findings related to the oral rehabilitation process were identified: Emotional loneliness prior to the oral rehabilitation, appreciation of a person‐centred care approach during treatment, and a lack of post‐rehabilitation initiatives. To secure oral health with older age, we suggest a long‐term, relational, both person‐ and people‐centred approach.

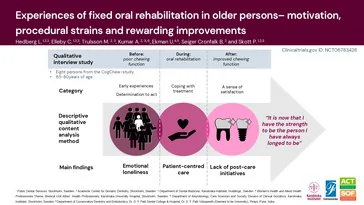

## 1 Introduction

A part of normal ageing, old age has been suggested to be a predominant cause of the acceptance of oral functional loss [1, 2, 3]. Age‐related structural and functional changes, systemic conditions, and medication‐induced xerostomia all contribute to a higher risk of deteriorating oral health [4]. Oral disorders are the most prevalent non‐communicable diseases worldwide, affecting almost half of the world's population, with dental caries and periodontitis being the most common [5]. Among older adults aged 60 years or older, one in four (23%) is estimated to be edentulous [5]. It may, however, be improved by oral rehabilitation. In addition, human epidemiological studies have shown associations between a lower number of teeth and increased functional health risks, including cognitive decline and frailty in older age [6, 7]. Moreover, a lower number of remaining teeth is strongly associated with reduced chewing ability and increased risk of malnutrition [8]. The physical consequences of tooth loss have also been described to affect a person's psychological well‐being and participation in social activities [9, 10, 11, 12, 13]. Research regarding the consequences of tooth loss and oral rehabilitation has mainly been explored using quantitative methods [14], while qualitative studies addressing issues concerning individualized treatment are rare. Even so, qualitative interview studies have been performed before, reporting improved perception of chewing and eating after oral rehabilitation [15, 16, 17]. With an ageing and growing population, demand for oral rehabilitation will rise. Therefore, a deeper understanding of older adults' needs can facilitate the whole individualized treatment trajectory with increased patient satisfaction.

In an ongoing intervention study with a longitudinal design, the Cognitive Change and Chewing Efficiency study (CogChew) [18, 19], patients received fixed oral rehabilitation after reduced chewing ability. These patients participated in comprehensive assessments of their neuropsychological status in addition to oral function at three months post‐rehabilitation. Further follow‐up assessments at 12 months post‐rehabilitation are planned. To further complement the quantitative data and self‐assessment questionnaires in this intervention study, a qualitative study was conducted to explore the patients' experiences of the oral rehabilitation. A subgroup of eight older adults from the intervention took part in this interview study, with the purpose of obtaining information and thereby understanding their experiences of extensive oral rehabilitation before, during, and after. This is, to our knowledge, the first time patients' perceptions of the whole process of extensive oral rehabilitation with chewing difficulties at inclusion have been investigated.

## 2 Method and Study Design

### 2.1 Research Ethics

The Swedish Ethical Review Authority approved this study on 2 October, 2024 (ref. nr 2024–04673‐01).

### 2.2 Research Team

The research team consists of seven researchers with multidisciplinary backgrounds, four senior dentists all with clinical dental research expertise (LH, CE, MT, AK, PS), a senior nurse and researcher with expertise in nursing science and qualitative methods (BS), and a clinical psychologist with expertise within neuroscience (UE).

### 2.3 Participants

The study employed consecutive sampling of all eligible participants following completion of extensive oral rehabilitation within a randomised intervention study conducted at the public dental services Eastmaninstitutet, Stockholm, Sweden. In all, ten participants were eligible to participate within the time span of the CogChew study. Two people were excluded due to long‐stay trips. In total, eight participants accepted participation, five women and three men. One of the women included served initially as a test person for the interview guide. All eight participants agreed to take part in the study after receiving oral and written information. Inclusion criteria: Persons ≥ 65 years, with lack of bilateral posterior occluding support zones according to the Eichner index <B2 [20, 21], who had completed oral rehabilitation with fixed prosthodontics, dental and/or implant‐supported, with four posterior occluding support zones according to the Eichner index >A2 [20, 21]. Exclusion criteria: Suspected cognitive impairment, defined as a Mini‐Mental State Examination (MMSE) [22] score < 25, depression, stroke ≤ 6 months before inclusion, severely impaired hearing or vision, or difficulties speaking, writing, or reading the Swedish language [18].

### 2.4 Study Design, Context, and Procedure

This qualitative interview study has an exploratory design with a descriptive approach utilising inductive manifest content analysis as described by Graneheim & Lundman (2004) [23]. This study conforms with the ENTREQ statement and COREQ guidelines for reporting qualitative studies. The study adopted a person‐centred approach (PCC) [24, 25], focusing on the persons' perceptions, experiences, and reflections following a period of oral rehabilitation [18]. After completing oral rehabilitation, information about the interview study was provided by the main author (LH) in connection with follow‐up testing in the CogChew study. One of the co‐authors (CE) contacted each person by phone or email to find out if they all agreed to participate in the study. After the person's decision and verbal consent, the time and place to meet for the interview were decided. Written consent was obtained at the time of the interview, with a copy provided to the participant. The interviews were conducted at a specialist dental care centre with five participants, at a specialist clinic for geriatric dentistry with one participant, and in the private homes of two participants.

The interviews focused on perceptions, experiences, and reflections before, during, and after the oral rehabilitation procedure. The questions concerned the participants' own observations regarding their previous and present life situation, well‐being (physical, mental, and social), and economic aspects. Examples of questions posed: *Can you please describe how your oral health was before the oral rehabilitation started? How would you describe the oral rehabilitation process? Could you please describe your experiences and life situation after the oral rehabilitation? When you think back, would you say that your experience corresponds to your thoughts and expectations before you started your oral rehabilitation?*

The interviews lasted between 60 and 80 min and were carried out by two of the co‐authors (BSC, CE), both females, equally sharing and alternating the responsibility for conducting the interviews and acting as moderators, respectively. The participants were informed that they could add or change information following the interview. They were also informed that they could withdraw from participating at any time without consequences. All interviews were tape‐recorded and transcribed word‐for‐word. Data were securely archived at the Stockholm public dental services.

Data saturation was continuously considered by the authors (CE, PS, LH, BSC) until saturation was determined, and no new information was provided during the interviews. The sample size emphasises the richness, depth, and variation of the data provided from the interviews.

### 2.5 Analysis

A qualitative content analysis with an inductive approach was performed [23]. The analysis included listening to each interview before the so‐called naïve reading (gaining an overall understanding of the content). This was followed by the identification of meaning units, i.e., single units of meaning regarded as an entity describing the participants' experiences related to their oral health and life situation before, during, and after the oral rehabilitation. The meaning units were then summarised and divided into codes, then grouped into four categories with sub‐categories (Table 1 illustrates the analytic process). The analysis was first conducted separately, then together by the authors. Three group discussions guided the authors as similarities and differences were debated regarding codes and categories before reaching an agreement. To lessen the influence of the authors´ pre‐understanding, the authors actively reconsidered and reflected upon this together and by themselves [26].

**TABLE 1:** Example of the analytic process.

Meaning unit	Code	Category	Sub‐category
“I did not want to see anyone in my spare time. Just because of my teeth, I was so ashamed”. (woman)	Coping strategy	Early experiences	Emotional loneliness
“Something, had to be done…the shame was terrible ”. (woman)	Decision to go through with the oral rehabilitation	Determination to act	Path to decision
“The only time it was painful was when they used the anaesthetic syringe, so I urged them to give me numbing salve. I did not suffer at all!” (man)	Feelings during the oral rehabilitation	Coping with the oral rehabilitation	Dealing with difficulties
“I feel that now it is done, I have done it. I accomplished it, I feel relieved and safe. It was something that had to be done, I knew that. I did not want to be toothless”. (woman)	Experiences after the oral rehabilitation	A sense of satisfaction	Experience of fulfilment

## 3 Results

### 3.1 Overall Findings

The findings portray eight unique narratives of a time before admission to oral rehabilitation, during, and after completed rehabilitation. The main findings show that the participants had their individual strategies to cope in everyday life before oral rehabilitation. A main strategy was to modify their food intake to easy‐to‐chew foods, but also change or even avoid social life. During the oral rehabilitation, a person‐centred approach by the specialist team was important for the participants to feel safe and acknowledged. It was described as helping ease worries and anxiety during the time of oral rehabilitation. It was important to be treated with respect and kindness during the whole process. This was considered essential for how they experienced the time they spent at the clinic. After the oral rehabilitation, the participants expressed a sense of uncertainty, a lack of both future dental plans and adherence to oral hygiene recommendations. The findings are presented in four categories: *Early experiences, Determination to act, Coping with the oral rehabilitation*, and *A sense of satisfaction*, including sub‐categories.

### 3.2 Early Experiences

Regardless of how long the participants had experienced oral problems, all described how they had their own strategies to manage everyday life.

#### 3.2.1 Strategies to Cope

The participants described strategies to cope as either conscious or subconscious. The most frequently described strategy was to consciously change the food intake and eating habits. An example was how they chose easy‐to‐chew food such as fish or soup. For one participant, another dimension of mealtimes was described as it caused feelings of worry:

“Mealtimes were tense for me… I worried, will I lose another tooth?”(man)

Other strategies mentioned were to hide or cover their teeth during social events or to avoid such events.

“I withdrew (from social events) without thinking about it.” (woman)

#### 3.2.2 Social Support

Support from family, friends, and neighbours was described as crucial. The time before deciding to accept oral rehabilitation was described as an emotionally difficult time.

“I am glad that you brought it up (comment to the interviewer). I was worried, very worried that they (grandchildren) would think that their Granddad looked different. But no… they did not bother”(man)

The participants described feelings of being supported and, in some cases, shielded by their family members and friends.

“I can't say that I had a social life. It was limited to my family… I just did not see other people at all” (woman)

Even though most participants spoke about the support they received from loved ones, it was described as difficult by one participant:

“It's hell… Without teeth the lips vanish from your face and to kiss your wife is not the same anymore…” (man)

#### 3.2.3 Emotional Loneliness

Poor and failing oral health was described as devastating. For some, life was influenced by their oral health to such an extent that they preferred to be on their own. This is to protect themselves from unpleasant remarks. One participant reflected on the impact it had on her life.

“I withdrew from socialising with friends in my free time, because of my teeth… When I look at the photos (of me) today, I looked awful, and I understand that I did not want to show myself” (woman)

Still, some described how they dealt with their feelings of being on their own as acceptable.

“I was not worried to be alone or left out” (woman)

### 3.3 Determination to Act

Many described that the decision to contact the specialist clinic was made after consideration with family and friends. Even so, some described it as challenging to find information about who to turn to.

#### 3.3.1 Path to Decision

Participants who visited their local dentist described that they were told about the necessity to contact a specialist dental clinic. If the participant acknowledged the advice, a referral was sent.

“He (the dentist) said that the only solution for me was to get tooth implants” (man)

Others described that they were approached by family members or friends.

“My daughter said, ´*Dad, the way you look, no woman would want you´*. It was in a way both an existential and ethical decision to make as I want to be a handsome man” (man)

As the participants were referred to the specialist clinic, they were asked if they would like to participate in a research study.

“I felt prioritised as I accepted to participate in a research study” (woman)

#### 3.3.2 Economic Conditions

A main concern that was described was the cost of the oral rehabilitation after receiving the information and estimate. The participants' economic ability varied, from their own savings to different means of loans or funds. As the cost of a completed rehabilitation was substantial, it was described as a predicament for some participants, while others had foreseen the cost and had savings.

“… One must have the means to do this (rehabilitation). I have saved money… I have always put money aside as this is something I have prioritised” (woman)

Some needed time to reflect and consider how they would solve it (the economy), while others were able to receive loans with an instalment plan.

“I got an estimate from the clinic, and I went home and thought to myself, *I must do this no matter what, I must solve it somehow*. I applied for a bank loan and got it, then called the clinic and said *Let's do it!*” (woman)

Two participants received funding from the local parish and the local community to cover the costs, while others had family members who helped with economic support.

“It was my daughter who paid for the treatment; I could never afford it” (woman)

“I thought to myself, *I don't know if I can make it (financially)*. I made it thanks to private funds, gratifications, and small savings…”. (woman)

### 3.4 Coping With Oral Rehabilitation

Managing life during the long rehabilitation process, approximately 12–18 months, was described as dealing with different difficulties and side effects. Verbal information regarding the oral rehabilitation was provided to all participants in line with standard clinical practice.

“Everyone explained exactly what would happen, so I knew” (woman)

Still, for some, the information was expressed as too difficult to comprehend or to fully understand.

“I had my daughter with me the whole time. Her memory is better than mine. Sometimes there was a lot of new information that I found difficult to comprehend, then she helped me” (woman)

#### 3.4.1 How to Handle Difficulties

Some participants described their difficulties and side effects as unbearable, even if they were expected. Others described various positive approaches to deal with their problems. Some even expressed the ability to predict what the oral rehabilitation might include.

“It was not nice, not nice at all. I was poorly the whole time. I was ill and not able to eat. My tongue was the problem as it was rubbing against knots from the stitches… causing an ulcer” (woman)

“Pain is something I am familiar with; it should hurt” (man)

However, most uttered that the time they spent in treatment appointments and the interlude between them was a time of enduring complications.

“I slept a lot and read books and watched movies. I knew it would take time, but it was okay…” (woman)

“I have a terrible gag reflex, and it was really problematic (during treatment)” (woman)

The oral rehabilitation procedure of extracting their teeth was for some expressed as existential. The loss of teeth was strongly connected to their identity.

“I grieved when they pulled my teeth out, even if they were bad. It hurts, not only the physical pain but also the psychological pain, to lose one's teeth. They have been a part of me my whole life and a part of my identity” (man)

Even with difficulties, all participants described the feeling of recognition, support, and security from the specialist team.

“They (the specialists) had good communication with me… it was great teamwork, me and them together. It meant a lot for me as a patient” (man)

### 3.5 A Sense of Satisfaction

In this last category, all participants described a sense of relief. All had completed their oral rehabilitation and uttered a sense of achievement and satisfaction with their own personal capability to act and to finish their oral rehabilitation.

“I sense a glimmer of hope…”. (woman)

“I feel whole again” (woman)

#### 3.5.1 Experiences of Fulfilment

The insight of being able to eat most foods again was expressed as their well‐being increased. One participant talked about the joy of being in a social context again, together with children and grandchildren during Christmas.

“The dentist did a great job, especially with the implant, but also regarding my general appearance. I am so pleased” (woman)

A sense of achievement was also uttered, including the ability to handle the economy, an earlier source of worry.

“It (the cost) has been worth it… I am worth it” (woman)

Not all had a positive experience with the oral rehabilitation. One participant described depressed symptoms for some time after the oral rehabilitation was completed.

“I was really depressed for some time, but it is better now… the treatment was difficult to deal with. It was hard” (woman)

#### 3.5.2 Plans of Future Dental Care

As the oral rehabilitation was completed, the participants received information regarding post‐rehabilitation care. Most participants described that they aimed to carry out the suggested procedure, but some were hesitant.

“The dentist told me that I need to use a high‐fluoride toothpaste and that it is important. I got a prescription… but it was too expensive, so I did not bother to get it” (woman)

The participants described that they were uncertain about how they should proceed and who to turn to (local dentist).

“I have not been in contact with my dentist, and I don't know if it is them or me who should make contact…” (man)

Instead, most participants referred to the one‐year follow‐up at the specialist clinic as the time they would get a dental check‐up.

“I have no plan really. I know that I have a one‐year follow‐up here… Apart from that, I have been told to contact a dental hygienist every six months… I'll see what I do about that” (man)

During the interviews, all participants expressed a sense of relief, satisfaction and fulfilment of being able to eat and socialise without worries, for some a lifelong desire.

“It is now that I have the strength to be the person I have always longed to be” (woman)

## 4 Discussion

This interview study aimed to broaden our understanding of older adults' experiences of oral rehabilitation. The interviews addressed questions concerning experiences before, during, and after the rehabilitation. Our findings suggest that during the oral rehabilitation process, the applied person‐centred care (PCC) method [25] was important to create a safe and respectful environment. Furthermore, strategies to deal with eating habits and social engagements were common. A main strategy concerning food intake was the shift towards easy‐to‐chew food, also described as a reason for changed social behaviour, contributing to feelings of emotional loneliness. Following the rehabilitation process, we also found that the participants were uncertain about their plans for continued dental care.

Old age has previously been described [1, 2, 3] to contribute to the acceptance of oral function loss as a part of normal ageing. Findings in our study suggest that the participants shared the objective of entitlement and self‐worth as they committed to oral rehabilitation, regardless of oral condition. These findings support the shift in social norms [3] with higher expectations and demand for improved oral function and aesthetics among older persons. This observation may be interpreted as a shift in stereotype attitudes and ageism.

### 4.1 Person‐Centred Care

A key finding in this study was the participants' experience of interacting with the specialist team. It was described in caring and comforting terms as they expressed that they were met with respect. The PCC approach is described as a holistic method to identify the person behind the diagnosis or disease, using an ethical foundation and underscores an empathetic interaction of trust between patient and staff as described by Ekman et al. [25]. We suggest that when personal attention was offered by the specialist team, it improved the interaction with the participants, subsequently consolidating a bond of trust. This holistic approach enables the definition of the individual's needs and specific wishes, allowing individualized treatment plans including mental wellbeing and social factors in addition to medical history. Mills et al. [27], showed that connection, attitude, communication, empowerment, and valued feelings were regarded as important perspectives when in dental treatment [27]. Relational skills by dentists have been greatly valued [27, 28] and are suggested to be essential in the care of older adults [29].

During the participants' rehabilitation, physical and physiological problems were accepted by most if they were anticipated in advance. Still, some experiences were unexpected and unpleasant, emphasising the importance of pre‐rehabilitation information. In our study, it became apparent that some participants found the information given hard to comprehend, including difficulties navigating and understanding the different oral rehabilitation procedures. This indicates the need for individualized support and understanding of a person's cognitive and coping ability. Although face‐to‐face communication has been proposed as a strategy to improve the understanding of different oral rehabilitation modalities [30], this must be considered as not enough in our study. Therefore, it ought to be the specialist team's endeavour to develop tailored strategies and to introduce more stringent actions, such as introducing motivational interviewing [31].

### 4.2 Emotional Loneliness

Emotional loneliness has been shown to correlate with depression among older adults, especially the oldest and people experiencing stress, decreased control, and anxiety personality traits (neuroticism) [32]. The participants in our study, with long‐term and poor oral health, reported emotional loneliness mainly connected to stigma and shame. These findings are supported by previous studies suggesting that poor oral health and tooth loss are associated with feelings of social distress [9, 13, 16, 17, 33]. Emotional loneliness has also been described as the absence of deeper personal relationships, leading to isolation and emotional detachment from family and friends. Wolters et al. [34], describe emotional loneliness as connected to social isolation and social anxiety [34]. This supports our findings as social isolation and inactivity were present before and during oral rehabilitation. The participants described loneliness and isolation as difficult to endure, causing poor well‐being. This corresponds with other studies [35, 36] arguing that extensive tooth loss and depressive symptoms have a bidirectional association. However, visiting the specialist clinic long‐term during the oral rehabilitation was not considered difficult or strenuous. The PCC approach during the constant visits should therefore be considered an important facilitator to break down feelings of isolation. The participants in our study did express positive feelings after being rehabilitated; it was expressed as gaining a sense of empowerment through mastery and social resilience. To the best of our knowledge, the present study is the first to explore oral health and emotional loneliness in this regard. Further research is therefore required to explore this in depth.

### 4.3 Lack of Post‐Rehabilitation Plans

An unexpected and important finding in this study was the participants' absence of post‐rehabilitation plans. We found this a matter of concern as non‐compliance with regular follow‐up with a general dentist or dental hygienist was evident. This could compromise the participants' long‐term prognosis regarding their oral health and has been confirmed by earlier studies [37, 38, 39]. Lack of compliance in dental and health care has previously been described by Bischof et al. [31], who advised motivational interviewing as a means to promote the participants' adherence [31]. The underlying reason in our study remains unclear, but the factors described above are of particular interest. Other explanations could be: Cost of the follow‐up visits, lost contact with the general dental caregiver, treatment fatigue, and/or a sense of reduced urgency related to the specialist's one‐year follow‐up. We should also consider that older adults have been shown to overrate their oral function [2] and the longevity of the oral rehabilitation effects [30]. To conclude, special attention is needed to support the participants' need to re‐establish their contact with the initial dental clinic. Therefore, a rigorous structure regarding reporting back to the referring dentist from the specialist clinic is needed for post‐rehabilitation care. To facilitate this transition, discharge strategies in line with PCC have shown positive effects on patient engagement [40]. We therefore suggest a structured referral report from the specialist clinic concerning the patients' need for post‐rehabilitation care. In addition, the patient would receive a person‐centred discharge letter during the last consultation to assist patient literacy. Further, staff‐oriented PCC education should be considered as an asset to upgrade the staff's understanding of interpersonal aspects in general dental medicine and support PCC implementation at an organisational level [41].

A strength of this qualitative study is that the patients were already included in the main oral rehabilitation study [18]. To analyse data, a qualitative content method with a manifest inductive approach was used [23, 26], as our aim was to focus on how the patients described their experiences. To ensure trustworthiness of the results, credibility, transparency, dependability, and confirmability, strategies described by Graneheim & Lundman [26] were used. The exploratory design allowed patients with a broad spectrum of etiological causes to participate. This, too, is regarded as a strength in this study. In addition, this study contributes to the field of qualitative studies in this area of dentistry.

A potential limitation in the study is the retrospective approach and exclusion criteria, such as a lack of previous intellectual disabilities. This may have limited patients' cognitive interference with the stressful rehabilitation events. Another limitation that could also be a limitation is the varied and individualized oral rehabilitation strategies used. In the future, it may be better to investigate a homogeneous group of patients in this regard.

## 5 Conclusions

Our findings highlight the relationship between perceived emotional well‐being, oral function, and the demand for tailored support in older adults going through oral rehabilitation. An unexpected finding was the patients' lack of plans for their future post‐rehabilitation care, potentially jeopardising the long‐term prognosis. To secure oral health, it will require a long‐term, relational, and people‐centred approach addressing these interconnected factors. To the best of our knowledge, this is the first study that incorporates poor chewing ability before oral rehabilitation, including both tooth‐ and/or implant‐supported fixed prosthetics.

Future research in this area should address issues of concern presented in this study: Emotional loneliness, possible concerns regarding cognitive impairment, and strategies to assist the patients' ability to understand the information given regarding their oral rehabilitation.

## Author Contributions

**L. Hedberg:** conceptualization, writing – original draft, methodology, validation, visualization, writing – review and editing, formal analysis, project administration. **A. Kumar:** writing – review and editing. **C. Elleby:** conceptualization, investigation, writing – original draft, methodology, writing – review and editing, visualization, validation, formal analysis, project administration, supervision, data curation. **M. Trulsson:** writing – review and editing, funding acquisition. **P. Skott:** resources, supervision, formal analysis, writing – review and editing, visualization, validation, methodology, funding acquisition, conceptualization. **U. Ekman:** writing – review and editing. **B. Cronfalk Seiger:** visualization, validation, methodology, conceptualization.

## Funding

The study has been supported by grants from the Stockholm County Council and Karolinska Institute (SOF: Styrgruppen för odontologisk forskning) and the Sigrid de Verdier Memorial Research Foundation.

All authors have read and agreed to the published version of the manuscript.

## Disclosure

No AI system has been used in study design, transcription, analysis, or in the formulation of the results, interpretations, conclusions and scientific arguments. The authors take full responsibility for the integrity and validity of the submitted manuscript.

## Conflicts of Interest

Abhishek Kumar is an Associate Editor of the Journal of Oral Rehabilitation and a co‐author of this manuscript. He had no involvement in the editorial process. All other authors report no conflicts of interest. The authors alone are responsible for the content and writing of the paper.

## Data Availability

Data sharing not applicable to this article as no datasets were generated or analysed during the current study.
